# Examination of the association of physical activity during pregnancy after cesarean delivery and vaginal birth among Chinese women

**DOI:** 10.1186/s12978-018-0544-1

**Published:** 2018-05-24

**Authors:** Xin-Ying Qi, Yan-Ping Xing, Xue-Zhen Wang, Feng-Zhen Yang

**Affiliations:** The Second Department of Obstetrics, Cangzhou Central Hospital, NO.16 Xinhua Road, Yunhe District, Cangzhou City, 061000 Hebei Province China

**Keywords:** Vaginal birth after cesarean delivery, Trial of labor after cesarean delivery, Physical activity, Exercise

## Abstract

**Background:**

The goal was to study whether higher physical activity can increase the success rate of Vaginal Birth after Cesarean Delivery (VBAC).

**Methods:**

We enrolled 823 patients with previous cesarean section delivery history (between January 2015 and December 2017) and measured their physical activity during pregnancy. A final number of 519 patients were included for the trial of labor after cesarean delivery (TOLAC). All patients signed informed consent forms.

**Results:**

We conducted bivariate analyses and identified that several variables were associated with successful VBAC: Prior history of vaginal birth (odds ratio [OR] 2.4, 95% CI 1.8–3.9); previous indication for primary cesarean delivery (OR 2.2, 95% CI 1.5–3.0); age younger than 40 years (OR 2.1, 95% CI 1.3–3.4); Weight gain less than 20 kg (OR 1.5, 95% CI 1.3–2.4); high pelvic/birth weight score (OR 1.4, 95% CI 1.1–2.0); no induction of labor (OR 1.9, 95% CI 1.4–2.8); and estimated prenatal fetal weight (OR 1.4, 95% CI 1.2–1.5). We also found that the bivariate association between physical activity and VBAC was significant (*p* = 0.002). In addition, there was higher odds of VBAC in women who had active physical activity of more than 150 min/week (adjusted OR 1.86, 95% CI 1.69–2.07). Lower odds of VBAC was associated with older age, weight gain during pregnancy, induction of labor, and having estimated prenatal fetal weight more than 3500 g.

**Conclusion:**

Physical activity during pregnancy may influence the success rate of VBAC in Chinese women. Future studies will be needed to prove the robustness of this association using more detailed exposure and outcome definitions.

## Plain English summary

Physical activity participation during pregnancy has beneficial effects for both the mother and baby. However, there is no report on whether higher physical activity can help normal vaginal child birth in women who had a previous C-Section child birth. Hence, we enrolled 823 patients (Han Chinese women) with previous cesarean section delivery history (between January 2015 and December 2017) and measured their physical activity during pregnancy. A final number of 519 patients were included for the trial of labor after cesarean delivery (TOLAC). All patients signed informed consent forms. We identified that several factors are associated with successful vaginal child birth in women who had a previous C-section. These factors include prior history of vaginal birth, previous indication for primary cesarean delivery, age younger than 40 years, weight gain less than 20 kg, high Bishop score, no induction of labor, and estimated prenatal baby weight. We also found that higher physical activity is associated with increased vaginal birth. We thus draw the conclusion that higher physical activity during pregnancy is associated with higher chance of vaginal birth in women who had a previous C-section child birth.

## Background

Cesarean section (CS) rate in China has been increasing dramatically. Particularly, the CS rates in 17 big cities in China were between 18.2 and 68.8%, with a median of 48.7% in 2008 [1]. The demand for second childbearing has been increasing recently in China due to the change of family planning policy. Hence, pregnant women face the decision of having an elective repeat cesarean delivery or attempting a vaginal birth after cesarean delivery (VBAC) [2]. However, the high rate of CS in China has brought critical concerns to the risk of maternal rupture of uterus during labor and delivery and other adverse outcomes associated with the trial of labor after cesarean delivery (TOLAC). Studies have shown that cesarean section after an unsuccessful TOLAC is associated with increased bleeding, postoperative infection, endometritis and other incidences [3–6]. and successful VBAC reduces morbidity compared to an elective repeat cesarean delivery [4, 7, 8]. Hence, successful VBAC is obviously more beneficial to the mother and child.

There is considerable evidence that physical activity participation during pregnancy including child and adult care giving, indoor household, recreational activities, and physical exercise has beneficial effects for both the mother and fetus [9–12]. Studies have shown that physical activity may help prevent gestational diabetes, pre-eclampsia, support healthy weight, and improve mental health for the mother [9, 12]. Regular physical activity may also help maintain cardiovascular fitness during pregnancy and may positively impact postpartum recovery [13–15]. Fetal benefits include reduced stress response and healthier birth weight [16]. Researches have been done to determine if exercise during pregnancy affects the mode of delivery. Some studies have found no relationship between mode of delivery and maternal activity during gestation [17–19]. Other researches, however, have found participation in exercise while pregnant is associated with lower rates of cesarean section and less complications [20]. Nonetheless, few studies have been conducted to examine the effects of physical activity on the success rate of VBAC. Hence, we designed this study to measure the physical activity during the pregnancy of second child after a previous cesarean section delivery. We aimed to study whether higher physical activity can increase the success rate of VBAC.

## Methods

### Study participants

A total of 823 eligible pregnant women with previous cesarean section delivery history were recruited between January 2015 and December 2017 in the Department of Obstetrics at Cangzhou Central Hospital, Hebei Province, China. Our facility is the major health facility located at the urban region of Cangzhou City and serves both the urban and surrounding rural areas of Cangzhou City. Women were eligible to participate if they were: 1) at least 18 years of age, 2) between 8 and 16 weeks pregnant, 3) owned a smartphone with text message capability, 4) had regular access to a computer, 5) willing to provide a cell phone number to receive text messages, and 6) willing to wear a physical activity monitor throughout their pregnancy. Women were ineligible if they were: 1) considered a high risk pregnancy (defined by The ACOG’s Position Statement on Exercise During the Pregnancy and Postpartum Period [21], and 2) limited to physical activity or instructed by a physician to limit physical activity. Eligible pregnant women were further enrolled for TOLAC based on the following eligibility criteria:

Inclusion criteria:

1. Did not show the same indications for cesarean section as in previous transverse uterine incision, and did not show a new indication for cesarean section
2. A singleton pregnancy
3. The previous cesarean section used transverse incision, and was more than 18 months ago
4. No history of maternal rupture of uterus during labor and delivery
5. No history of repeated uterine injuries, such as uterine perforation, myomectomy, uterine rupture repair
6. Gestational age was more than 35 weeks, but no more than 40 weeks
7. No serious complications of pregnancy and surgical complications (e.g., severe bleeding, infection, etc)
8. Ultrasound examination shows continuous lower uterine segment scar
9. Willing to accept TOLAC and understand the advantages and disadvantages of vaginal delivery and cesarean section
10. Signed patient consent form

Exclusion criteria:

1. The last cesarean section used classical cesarean section incision, uterine t-shaped incision or incision unknown
2. The previous cesarean surgical indications still exist or there are new indications for cesarean section
3. Cephalopelvic disproportion
4. Signs and symptoms that raise suspicion of uterine rupture
5. Uterine tenderness reported during prenatal visit
6. Ultrasonic examination during prenatal visit found the following conditions: a) lower uterine scar defect or uneven thickness; b) lower uterine muscle fiber loss; c) the amniotic membrane from the thin section of the uterus to the anterior abdominal wall prolapse of the bladder; d) fetal movement visible
7. Previous cesarean section had infection or uterine diverticulum
8. Surgical complications and obstetric complications in last pregnancy
9. More than 1 history of cesarean section
10. Postterm pregnancy

### Collection of physical activity data

After the eligibility of subjects were confirmed, all subjects were requested to sign a consent form, complete a demographic questionnaire (e.g., age, race, ethnicity, income, education, number of chronic disease conditions, preferred number of days of wearing Fitbit Flex per week, and gestational age at enrollment), and report physical activity using the Modifiable Activity Questionnaire, Physical activity was measured weekly from entry into the study (8–16 weeks pregnant) until the end of the pregnancy (36–40 weeks). Fitbit Flex (Shanghai, China) was commercially available and was provided to each participant at no cost by study personnel upon receiving signed consent form. Instructions to wear and use the Fitbit Flex were also provided to the participants by study personnel during the prenatal visit when the consent form was signed. Specifically, participants were instructed to wear the Fitbit on their non-dominant wrist throughout pregnancy, 24 h daily (except during showers or swimming). Fitbit was instructed to switch to “Sleep” mode when sleeping or taking a nap based on the manufacture’s manual. The Fitbit has been shown to be valid measure of steps under laboratory conditions [22, 23]. Fitbit describes fairly active minutes to represent activities occurring at more than 3.0 metabolic equivalents (METs) and very active minutes at more than 6.0 METs [24]. For purposes of data analysis, we combined “fairly active” and “very active” in an “active time” category. During the study, Fitbit data were blinded to the participants, and only study personnel had access to Fitbit online account. Adequate instructions were given to participant to make sure compliance of participants to syncing their Fitbit and were notified by text message if they hadn’t synced the Fitbit within the last five days.

### Trial of labor after cesarean (TOLAC)

A total of 304 patients failed to comply with using Fitbit (i.e, missing at least one weekly data; *n* = 45), changed their decisions not to accept TOLAC during study (*n* = 239), had newborn with anomalies (*n* = 6), or had missing cervical examination data (*n* = 14). These patients were excluded from final analysis. As a result, a total of 519 patients were included in the final data analysis in this study. All cesarean and vaginal deliveries were performed in the Department of Obstetrics at Cangzhou Central Hospital, Hebei Province, China. Gestational weight gain was calculated by subtracting pre-pregnancy weight from admission weight at delivery admission. Women admitted for TOLAC are managed by certified midwives and decisions for eligibility for TOLAC, induction of labor or oxytocin augmentation and mode of delivery were taken by board certified obstetricians. Induction of labor was performed either by an ATAD balloon, artificial rupture of membranes or oxytocin ripening of the cervix. Enrolled patients were identified by patient TOLAC request documented by the labor and delivery nurse at admission and the signed patient consent form. Medical records of patients undergoing TOLAC were used to collect demographic and obstetric data. Recorded obstetric variables included mode of delivery, use of oxytocin, preeclampsia or eclampsia, small-for-gestational-age neonate, gestational diabetes, cervical examination and indication for cesarean delivery during admission when delivery occurred. Rice pelvimeter was used to measure the pelvic area. The Bishop score [25] was calculated using the first digital cervical examination at the time of admission.

### Statistical analysis

A three-level variable describing physical activity during pregnancy was created, defined as less than 60 min of active time per week, 60–149 min of active time per week, and 150 min of active time per week or more. Number and percentages of the study variables were determined. Bivariate analyses were performed to test the association between the independent variables and the outcome variables using chi-square tests. Multivariable logistic regression was then used to model the association between physical activity during pregnancy and VBAC during TOLAC. In the model, we controlled for variables and covariates known to be associated with VBAC, including age, race/ethnicity, gestational weight gain, delivery history and pre-pregnancy diabetes or hypertension. All analyses were performed using SAS Version 9.3 (SAS Institute, Inc., USA).

## Results

The demographic characteristics of women undergoing TOLAC are shown in Table 1. The rates of preeclampsia, gestational diabetes, and small-for-gestational-age neonates were relatively low. Seven of those women undergoing TOLAC were diagnosed with uterine rupture. All ruptures occurred in the setting of spontaneous labor. All ruptures were discovered at 5–6 cm of dilation. None of the ruptures required a hysterectomy. All of these newborns survived without apparent neurologic impairment. The rates of other obstetric complications in the women with a successful VBAC (*n* = 367) are as follows: shoulder dystocia in 3.3% (*n* = 12; 95% CI 1.1–2.4); third or fourth-degree laceration in 9.1% (*n* = 33; 95% CI 6.4–10.8); and operative vaginal delivery in 11.2% (*n* = 41; 95% CI 8.9–11.6).

**Table 1:** Demographic characteristics of women undergoing trial of labor after cesarean delivery

Demographic characteristic	Successful VBAC (n = 367)	Failed VBAC (*n* = 152)	*P*
Maternal age (y)	27.8 ± 4.9	35.6 ± 4.9	.011
Gestational age at delivery (wk)	38.8 ± 1.7	38.5 ± 1.9	.65
35–38 (wk)	198 (53.9)	77 (50.7)	
38–40 (wk)	169 (46.1)	75 (49.3)	
Weigh Gain During Pregnancy			<.001
Less than 15 kg	19 (5.1)	15 (9.6)	
15–20 kg	292 (79.8)	95 (62.3)	
More than 20 kg	56 (15.1)	42 (28.1)	
Marital status			.37
Married	342 (93.2)	143 (94.1)	
Single	13 (3.4)	3 (1.9)	
Divorced or separated	6 (1.7)	3 (1.9)	
Unknown	6 (1.7)	3 (1.9)	
History of vaginal delivery	143 (38.9)	29 (19.2)	<.001
Previous indication for primary cesarean delivery	37 (10.1)	41 (26.9)	<.001
Induction of labor	80 (22.0)	114 (75.0)	.019
Maternal preeclampsia or eclampsia	12 (3.4)	6 (3.8)	.69
Maternal gestational diabetes	18 (5.1)	9 (5.7)	.21
Small-for-gestational-age neonate	12 (3.4)	6 (3.8)	.73
Pelvic measurement (cm)			
Sacral shame external diameter	19.3 ± 1.4	17.4 ± 1.5	.042
Diagonal conjugate	13.4 ± 0.7	11.5 ± 0.8	.043
Transverse outlet	9.5 ± 0.4	7.9 ± 0.4	.039
Posterior sagittal diameter of pelvic outlet	17.7 ± 1.5	14.8 ± 1.6	.022
Anteroposterior diameter of pelvic outlet	12.6 ± 0.8	10.7 ± 0.6	.039
Estimated prenatal fetal weight (g)	3108 ± 466	3759 ± 521	.042

We conducted bivariate analyses using chi-square tests and identified that several variables were associated with successful VBAC: Prior history of vaginal birth (odds ratio [OR] 2.4, 95% CI 1.8–3.9); previous indication for primary cesarean delivery (OR 2.2, 95% CI 1.5–3.0); age younger than 40 years (OR 2.1, 95% CI 1.3–3.4); Weight gain less than 20 kg (OR 1.5, 95% CI 1.3–2.4); Higher Bishop score (OR 1.4, 95% CI 1.1–2.0); no induction of labor (OR 1.9, 95% CI 1.4–2.8); and estimated prenatal fetal weight (OR 1.4, 95% CI 1.2–1.5). We also found that the bivariate association between physical activity and VBAC was significant (*p* = 0.002; Table 2). In the multivariable model estimating cesarean delivery (Table 3), there was higher odds of VBAC in women who had active physical activity of more than 150 min/week (adjusted OR 1.86, 95% CI 1.69–2.07). Lower odds of VBAC was associated with older age, weight gain during pregnancy, induction of labor, and having estimated prenatal fetal weight more than 3500 g.

**Table 2:** Baseline characteristics of pregnant women and prenatal physical activity

	Total sample *N* = 519	Prenatal physical activity (minutes/week)			*p*-value*
		< 60 *N* = 134 (25.9%)	60–149 *N* = 216 (41.6%)	≥150 *N* = 169 (32.5%)	
Maternal age (y)					< 0.001
18–24	132 (25.5)	32 (24.2)	47 (21.7)	53 (31.5)	
25–29	206 (39.7)	55 (40.9)	94 (43.5)	57 (33.8)	
30–40	181 (34.8)	47 (34.9)	75 (34.8)	59 (34.7)	
Weigh gain during pregnancy					< 0.001
Less than 15 kg	368 (70.9)	97 (72.2)	162 (74.9)	109 (64.6)	
15–20 kg	93 (17.9)	23 (17.6)	34 (15.7)	36 (21.4)	
More than 20 kg	58 (11.2)	14 (7.5)	20 (9.4)	24 (14.0)	
Delivery history					0.001
Vaginal delivery	86 (16.4)	23 (16.2)	30 (14.0)	33 (19.5)	
Previous indication for primary cesarean delivery	138 (26.6)	38 (28.7)	54 (25.2)	46 (27.2)	
Induction of labor	295 (56.8)	74 (55.1)	131 (60.9)	90 (53.3)	
Estimated prenatal fetal weight (g)					< 0.001
> 3500 g	281 (54.1)	79 (58.6)	121 (56.1)	81 (48.0)	
< 3500 g	238 (45.9)	55 (41.4)	95 (43.9)	88 (52.0)	
VBAC	367 (70.7)	78 (58.2)	146 (67.6)	143 (84.6)	0.002

*chi-square test comparing women by prenatal physical activity level

**Table 3:** Logistic regressions modeling effect of prenatal physical activity on VBAC [adjusted odds ratios (95% confidence intervals)]

	VBAC *N* = 367
Prenatal physical activity	
< 60 min/wk	Reference
60–149 min/wk	1.09 (0.89–1.34)
150+ min/wk	1.86 (1.69–2.07)*
Maternal age (y)	
18–24	Reference
25–29	1.12 (1.02–1.30)
30–40	0.83 (0.73–0.94)*
Weigh gain during pregnancy	
Less than 15 kg	Reference
15–20 kg	1.04 (0.95–1.22)
More than 20 kg	0.79 (0.68–0.92)*
Delivery history	
Vaginal delivery	Reference
Previous indication for primary cesarean delivery	0.96 (0.77–1.22)
Induction of labor	0.72 (0.61–0.91)*
Estimated prenatal fetal weight (g)	
> 3500 g	Reference
< 3500 g	1.55 (1.30–1.85)*

Models are adjusted for age group, race/ethnicity, gestational weight gain, delivery history and pre-pregnancy diabetes or hypertension

^*^p < 0.05

## Discussion

Previous studies in Norwegian have shown that, regular exercise and high-impact exercises during pregnancy are associated with reduced risk of having an acute cesarean delivery in first-time mothers, compared with nonexercisers [26]. Similarly, a study in Spain showed that a supervised program of moderate-intensity exercise performed throughout pregnancy was associated with a reduction in the rate of cesarean and instrumental deliveries [27]. Furthermore, a study in the US confirmed that physical activity of 150 or more minutes per week was associated with reduced odds of cesarean delivery compared with less than 60 min per week [28]. Adding to the literature, our study first examined the association of physical activity during pregnancy and success rate of VBAC. While the body of literature in this field shows that VBAC rates varies widely between countries [29], previous studies together with ours suggest that exercise during pregnancy may be associated with a reduced risk of cesarean delivery in diverse populations. Thus, further studies incorporating diverse populations are warranted to validate and extend our findings.

In the present study, about 70.7% (367/519) of patients had successful VBAC with one previous CS. In these patients, most of them had spontaneous vaginal delivery (i.e., no induction labor). The successful rate is slightly higher compared with the results found in other studies, which showed that success rate of VBAC ranging from 60.0–80.0% [30, 31]. The relatively similar success rate of VBAC found in the present study may be due to the careful selection of patients based on our inclusion and exclusion criteria and the application of clinical evidences in the management of these patients. The present study also revealed that the chance for successful VBAC in women with prior vaginal delivery was greater than those with no history of prior vaginal delivery. This result is consistent with previous reports that prior vaginal delivery is the strongest predictor of successful VBAC [32, 33]. Furthermore, we also found that increasing maternal age is associated with failed VBAC. However, we did not observe the effects of gestational age on the success rate of VBAC. In contrast, we demonstrated that weight gain during pregnancy has a major effect on the success rate of VBAC. Specifically, weight gain more than 20 kg was associated with increased failure rate of VBAC.

We identified seven variables to be independently associated with successful VBAC. These are prior history of vaginal birth, no previous indication for primary cesarean delivery, age younger than 40 years, weight gain less than 20 kg. high Bishop score, no induction of labor, and estimated prenatal fetal weight. It should be noted that a modified Grobman’s model that is supplemented with estimated fetal weight improved the AUC in Chinese population, suggesting estimated fetal weight is an important factor predicting VBAC in Chinese women. As demonstrated in a number of previous studies that regular exercise was associated with lower birth weights [34–37] and maternal weight gains [38–41], it seems possible that higher physical activity due to exercise may play a role in enhancing VBAC by reducing birth weights and controlling maternal weight gains. Hence, future studies will be needed to prove the robustness of the association between physical activity during pregnancy and success rate of VBAC using more detailed exposure and outcome definitions. If time and amount of exercise is proven to be a key factor associated with the success rate of VBAC, it will be possible to explore etiologies and conduct intervention in the future. If the association proves causal, encouraging pregnant women to increase certain type of physical exercise could represent a low-cost, low-risk approach to reduce the rising CS rates, to which repeat CS contributes largely. The strength of this study is that physical activity was determined by objective measurement rather than self-report. However, limitations of our study exist. The physical activity varied throughout pregnancy, and we did not assess pre-pregnancy physical activity. Hence, we cannot assess how the amount of pre-pregnancy physical activity may affect VBAC. In addition, our obstetric population is highly homogeneous (mostly Han Chinese, married), which may increase the performance of our model when compared with previously reported models. Hence, the generalizability of our model to more heterogeneous and high-risk populations may be limited. Like any model assessing VBAC success rate, our model dose not account for many other factors, including physician counseling, patient preference, or labor management. In fact, there are still many women who chose an elective repeat cesarean delivery would be considered good candidates for TOLAC. Thus, future studies would be important to identify which factors impact most on women accepting or declining trial of VBAC (e.g. patient information, previous labor experiences, desired family size, understanding the risk analysis during counseling, hospital sitting, or cost effect). There are many well-established benefits to exercising during pregnancy both to the fetus and the mother.

## Conclusion

In this study, we have reported that higher physical activity during pregnancy is associated with higher odds of VBAC. However, reported levels of physical activity during pregnancy are low, and efforts to disseminate and implement the recommended guidelines are needed. Further, future studies will be needed to prove the robustness of the association between physical activity during pregnancy and success rate of VBAC using more detailed exposure and outcome definitions in diverse populations and determine whether the time and amount of maternal exercise are associated with the success rate of VBAC. If the association proves causal, encouraging pregnant women to increase certain type of physical exercise could represent a cost-effective approach to reduce the CS rates.

## Abbreviations

TOLAC: trial of labor after cesarean delivery
VBAC: Vaginal Birth after Cesarean Delivery
